# Comparing the Prognostic Value of Quantitative Response Assessment Tools and LIRADS Treatment Response Algorithm in Patients with Hepatocellular Carcinoma Following Interstitial High-Dose-Rate Brachytherapy and Conventional Transarterial Chemoembolization

**DOI:** 10.3390/cancers17081275

**Published:** 2025-04-09

**Authors:** Robin Schmidt, Christopher Rueger, Han Xu, Yubei He, Emine Yaren Yilmaz, Luisa Heidemann, Ornela Sulejmani, Yu Liu, Lasse Noack, Friederike Hesse, Richard Ruppel, Sara A. Abosabie, Charlie Alexander Hamm, Tobias Penzkofer, Bernhard Gebauer, Lynn Jeanette Savic

**Affiliations:** 1 Department of Radiology, Corporate Member of Freie Universität Berlin and Humboldt-Universität zu Berlin, Charité-Universitätsmedizin Berlin, Augustenburger Platz 1, 13353 Berlin, Germany; 2 Experimental Clinical Research Center (ECRC) at Charité-Universitätsmedizin Berlin and Max-Delbrück-Centrum für Molekulare Medizin (MDC), Robert-Rössle-Straße 10, 13125 Berlin, Germany; 3 Berlin Institute of Health at Charité-Universitätsmedizin Berlin, Charitéplatz 1, 10117 Berlin, Germany

**Keywords:** hepatocellular carcinoma, locoregional therapies, interstitial brachytherapy, transarterial chemoembolization, response assessment, LIRADS Treatment Response Algorithm

## Abstract

**Background/Objectives:** The aim of this study was to investigate the prognostic value of established response assessment tools for hepatocellular carcinoma (HCC) treated with high-dose-rate interstitial brachytherapy (iBT) alone or with transarterial chemoembolization (cTACE). **Methods:** (Non-)responders were categorized using size-based RECIST 1.1 and WHO criteria, enhancement-based mRECIST and EASL criteria, and the LI-RADS Treatment Response Algorithm (LR-TRA). The outcomes were the overall survival (OS), progression-free survival (PFS), and time to progression (TTP). The statistics used included Fisher’s exact test, a *t*-test, the Mann–Whitney-U test, and a Kaplan–Meier analysis. The median OS, PFS, and TTP were higher in patients following iBT (26.3, 9.1, and 13.0 months) than following cTACE/iBT (23.3, 7.6, and 9.2 months). **Results:** The enhancement-based criteria identified more responders and predicted PFS and TTP better compared to the size-based criteria. At two months, the cTACE/iBT responders showed improved PFS (mRECIST and EASL: 11.3 vs. 2.3 and 11.0 vs. 2.3, *p* < 0.01) and TTP (mRECIST and EASL: 11.9 vs. 2.4 months, *p* < 0.01) by the enhancement-based criteria. An EASL assessment at five months predicted improved survival following both cTACE/iBT (PFS: 11.9 vs. 5.1 months, *p* = 0.03; TTP: 12.4 vs. 5.0, *p* < 0.01) and iBT (11.1 vs. 5.1 months, *p* = 0.04; 13.0 vs. 5.3, *p* < 0.01). The LR-TRA showed OS benefits at five months for cTACE/iBT responders. Size-based criteria were not prognostic. **Conclusions:** Extending follow-up post-iBT or post-iBT/cTACE may improve responder stratification and prognostication.

## Simple Summary

The majority of patients with hepatocellular carcinoma are suitable candidates for minimally invasive locoregional therapies. While these liver-directed and tumor-orientated guideline-approved therapies are widely distributed, a standardized assessment of treatment response is lacking in cases of interstitial irradiation using high-dose-rate brachytherapy and combinations of brachytherapy with prior transarterial chemoembolization for the treatment of large, hypervascularized, and therefore potentially more aggressive tumors. While evaluating the most common treatment response measurement tools, this study found that size-based criteria had a lower prognostic value compared to enhancement-based tools. Our findings suggest that, besides the utilized tools, the appropriate time of response measurement will reliably distinguish good from poor responses, which may improve personalized surveillance strategies and may improve prognostication.

## 1. Introduction

Hepatocellular carcinoma (HCC) is the sixth most common cancer worldwide and the third leading cause of cancer-related death worldwide [1]. In patients with unresectable HCC, locoregional therapies (LRTs) are the guideline-approved standard of care, including ablation and embolization therapies for early- and intermediate-stage disease [2,3]. Recommendations by the European Society of Medical Oncology (ESMO)’s treatment guidelines for HCC as well as growing evidence suggests that CT-guided interstitial high-dose-rate brachytherapy (iBT) can be used interchangeably compared to thermal-energy-based ablation techniques such as microwave (MWA) or radiofrequency ablation (RFA) with the added benefits of treating larger lesions while avoiding injury to adjacent bile ducts and blood vessels [4,5]. iBT comprises interstitial radiation via the application of a beta-emitting iridium-192 source through a CT-guided catheter placement. Due to precise 3D radiation planning and the rapid dose drop outside the target tissue, brachytherapy allows for the ‘inside-out’ application of a very high radiation dose to the target volume in a single fraction (>100 gray in central tumor parts). Moreover, iBT can be combined with conventional transarterial chemoembolization (cTACE), particularly in patients with larger liver tumors up to 5 cm, for which thermal ablation alone might be insufficient to achieve complete treatment, and thus may result in higher rates of local recurrence [6].

While iBT induces gradual tumor necrosis, fibrotic scars, and the subsequent shrinkage of the tumor over time, the cTACE-induced effects are exhibited more immediately. Following the occlusion of the tumor-feeding arteries by the injection of ethiodized oil, chemotherapeutic agents, and embolic beads or foam, the tumor is devascularized, causing an early onset of necrosis via cytotoxic and ischemic effects.

Thus, therapeutic effects on postinterventional imaging may vary among different types of ablation and embolization therapies [7]. To evaluate tumor response with computed tomography (CT) or magnetic resonance (MR) imaging, uni- or bidimensional measurements are widely accepted and validated assessment tools. Size-based techniques such as the unidimensional Response Evaluation Criteria in Solid Tumors (RECIST) and the bidimensional World Health Organization (WHO) criteria measure the alteration in the largest axial diameter or area of target lesions, respectively. In contrast, enhancement-based techniques such as the unidimensional modified (m)RECIST and bidimensional European Association for the Study of the Liver (EASL) criteria were designed to quantify changes in the largest enhancing tumor diameter or area, respectively, particularly in hypervascularized tumors following LRT [8]. Moreover, the Liver Imaging Reporting and Data System (LI-RADS) Treatment Response Algorithm (LR-TRA) was introduced to overcome those linear definitions of (m)RECIST, the WHO, and the EASL while defining postinterventional tumor viability as a more complex pattern beyond alterations in size and enhancement [9]. In the latest update from early 2024, the 2018 version of the LR-TRA’s definition of tumor viability was expanded according to the respective LRT-induced mechanisms by including two separate algorithms for radiation- and non-radiation-based LRT [10].

Due to overall heterogeneity in tumor responses following LRTs, there is a significant clinical need for guidelines on response criteria for each treatment modality. While experts agree that response to cTACE should be measured using enhancement-based tools, the role of the LR-TRA in this context remains unclear, although some studies reveal potentially increased sensitivity in detecting viable tumor masses post-cTACE [11]. Additionally, interpreting the effects of ablation combined with prior embolization via cTACE is challenging [12]. Albeit the LR-TRA radiation core includes major irradiation-based therapies, there are still no recommendations for assessing tumor response for patients with HCC following iBT. Therefore, this study aims to compare established quantitative response assessment techniques and evaluate their prognostic value for survival in patients with HCC following iBT, both as a single treatment and in combination with prior cTACE.

## 2. Materials and Methods

### 2.1. Study Cohort, Design, and Endpoints

This retrospective observational single-center study was approved by the local institutional review board of ‘Charité-Universitätsmedizin Berlin’. Informed consent was waived due to the retrospective study design. The study protocol conforms to the ethical guidelines of the Declaration of Helsinki, as reflected in the prior approval by the institution’s human research committee. In total, 95 patients with a primary diagnosis of unresectable HCC who received either iBT (*n* = 48) or iBT in combination with cTACE one day prior to iBT (*n* = 47) between 01/2016 and 12/2017 were included in this study, allowing for an adequate follow-up period. Decisions on iBT alone or combined iBT ablation with cTACE as definitive treatment concepts were made by the institutional multidisciplinary tumor board (MTB) and at the discretion of the interventional radiologist. While iBT is considered to be the institute’s standard local ablation technique for the treatment of liver malignancies, combined iBT ablation with prior cTACE was considered in very large and highly vascularized lesions with the intent of increased treatment efficacy. All lesions were treatment-naïve to minimally invasive therapies. The primary HCC diagnosis was confirmed according to LI-RADS catalog or according to histopathology following a core needle biopsy.

The primary study endpoint was the comparison of patients’ survival when they were stratified as responders vs. non-responders according to RECIST, mRECIST, the WHO, the EASL, and LI-TRA radiation core assessed two and five months post-LRT. Parts of the study’s cohort have been included in analyses from original research published elsewhere [13].

### 2.2. Imaging and Clinical Data Retrieval

Baseline contrast-enhanced MR scans were obtained within 30 days prior to therapy and follow-up MR scans were acquired two months post-LRT, then every three months for the first year and every six months for the following years. Briefly, the MR protocol amongst T2-weighted sequences and diffusion weighted imaging included breath-hold unenhanced and contrast-enhanced T1-weighted imaging (VIBE and in-phase/opposed-phase FLASH) using a hepatocyte-specific contrast agent (Primovist, Bayer, Germany) including arterial, portal venous, venous, and hepatobiliary phases (15, 50, 90 s, and 20 min after contrast administration). MRI scans were either acquired by 1.5-Tesla scanners (Avanto and Aera) or a 3-Tesla scanner (Skyra, all Siemens, Erlangen, Germany) using an eight-channel body phased-array coil. Standard sequences were acquired in the axial plane covering the entire liver with 60–72 slices and with an adjusted field of view of 255–300 mm × 340–400 mm × z-axis. Images were evaluated with Visage PACS client version 7 (Visage Imaging). Additionally, medical records were reviewed using the oncologic database “Giessener Tumordokumentationssystem” to calculate the survival measures and progression patterns.

### 2.3. Response Assessment Measurements

Tumor response was assessed according to RECIST 1.1, mRECIST, the WHO, the EASL, and LR-TRA v2018 and v2024 radiation core criteria as they are described in detail elsewhere. Briefly, RECIST 1.1. and WHO were assessed using venous- and/or hepatobiliary-phase T1-weighted sequences and measurements that included the largest uni- and bidimensional diameter of viable tumors or post-therapeutic necrotic masses. mRECIST and the EASL were assessed using arterial-phase T1-weighted sequences and measurements that included the largest uni- and bidimensional diameter of enhancing viable tumors or post-therapeutic enhancing masses. Response assessment according to LI-TRA included evaluation on contrast-enhanced T1-weighted sequences as well as T2-weighted and diffusion weighted imaging regarding LI-TRA equivocal or non-progressing categories. Measurements were qualitatively performed [8,9,10]. Images were analyzed by two radiologists with four and nine years of experience in abdominal imaging, who did not allocate or perform the LRT and who were blinded to the respective survival outcomes. Patients were then stratified into responders (complete response, partial response, and LR-TRA non-viable) vs. non-responders (stable disease, progressive disease, LR-TRA viable, LR-TRA equivocal, and LR-TRA non-progressing, respectively). In this study, LR-TRA equivocal and non-progressing were categorized as analogous to stable disease and thus non-responders to simplify statistical analyses.

### 2.4. CT-Guided High-Dose-Rate Brachytherapy (iBT)

The detailed brachytherapy procedure is described elsewhere [14,15,16]. Briefly, patients obtained iBT under conscious sedation (midazolam and fentanyl) and with local anesthesia (lidocaine). Under CT-fluoroscopic guidance, a 6F angiographic sheath was inserted into the lesion, followed by a closed-end 6F brachytherapy catheter (Primed, Halberstadt, Germany) which was put through the sheath. The catheter arrow was depicted in relation to the tumor on a contrast-enhanced CT scan with primary slice thickness of 1 mm and reconstructed slice thickness of 5 mm. Further treatment planning was performed on a 3D radiation planning workstation (Brachyvision, Varian Medical Systems, Palo Alto, CA, USA) with the clinical target volume being manually drawn on planning scans. Intentionally, each lesion is thought to be ablated with a tumor-enclosing target dose of 20 gray (Gy) using an iridium-192 source (Gammamed 12, Varian Medical Systems). Adjacent structures at risk (e.g., stomach or duodenum) were manually marked, and their dosages were calculated as well. When necessary, overall dosage was modified according to Collettini et al. [6]. After treatment completion, the catheter was retracted and the puncture channels were sealed with resorbable thrombogenic material (Gelfoam, Pfizer Inc., New York, NY, USA).

### 2.5. Conventional Transarterial Chemoembolization (cTACE)

cTACE was performed under conscious sedation as previously described [17]. Intraarterial access was established through the common femoral artery. The tumor-feeding arteries were catheterized using a microcatheter and a microcatheter with guidewires. Once tumor blush confirmed the segmental or sub-segmental catheter placement, an emulsion comprising 5 cc of chemotherapy (50 mg doxorubicin in 2.5 cc and 1 mg of mitomycin-C in 2.5 cc) and 10 cc of ethiodized oil (Lipiodol; Guerbet, Villepinte, France) mixed in a ratio of 1:2 was slowly injected into the tumor site. Under digital subtraction angiographic control, the amounts of the mixture administered were titrated to the tumor burden followed by injection of Gelfoam (Pfizer Inc., New York, NY, USA) until blood flow stasis as the angiographic endpoint. Furthermore, pre- and post-procedural cone beam CT scans were frequently performed to assess tumor vascularization and Lipiodol distribution [18].

### 2.6. Completed Treatment Cycles

Sequential treatment cycles were routinely performed in patients with multifocal or large lesions at baseline with the intent to avoid adverse effects from tumor lysis or to reduce cumulative puncture risk. Finally, a treatment cycle was considered completed when all target lesions determined at baseline had been completely irradiated with the target dose of 20 Gy by iBT and/or when the Lipiodol following cTACE was homogenously distributed within the whole tumor mass.

### 2.7. Survival and Tumor Progression Measures

Overall survival (OS) was defined at the patient level as the time between completed treatment cycle and the date of death. Patients were censored in the event of eventual orthotopic liver transplantation (OLT), if they received additional LRT on the target lesion, or if they were alive at the end of follow-up.

Progression-free survival (PFS) was defined at the patient level as the time between a completed treatment cycle and death or the occurrence of tumor progression. Patients were censored at the respective time point in the event of OLT, if they received additional LRT on the target lesion, or if they were alive at the end of follow-up.

Time to progression (TTP) was defined at the tumor level as the time between completed treatment cycle and the occurrence of tumor progression. Patients were censored in the event of OLT, if they received additional LRT on the target lesion, or if they were alive at the end of follow-up.

In addition to overall PFS and TTP, two subtypes of progression patterns were analyzed. Local tumor progression (LTP) subtype was defined as an increase in the target lesion at any time after therapy, and intrahepatic distant recurrence (IDR) considered the occurrence of any new intrahepatic lesion that was not spatially associated with the primary target lesion.

New intrahepatic lesions that were not present at baseline but appeared during follow-up were considered tumor progression in the calculation of PFS (patient level). However, treatments of these new lesions were considered for the calculation of TTP (tumor level) as separate completed treatment cycles [19].

### 2.8. Statistics

The last follow-up data were collected on 23 December 2024. Descriptive statistics were reported as mean ± standard deviation (SD) or median and interquartile range (IQR), respectively. Statistical analysis included Fisher’s exact test, normality test, *t*-test, Mann–Whitney U test for comparing grouped patient and disease characteristics, Kaplan–Meier analysis, and log-rank test for the analysis of survival measures in regards to the treatment response criteria. Cox proportional hazard models of selected baseline patient, tumor, and disease characteristics and laboratory values were performed with OS, PFS, and TTP as dependent variables. Statistical significance was defined as a *p*-value < 0.05 for any analysis and <0.1 for regression analysis. Statistical analysis was performed using GraphPad Prism V9.0.0 (GraphPad Software Inc., La Jolla, CA, USA).

## 3. Results

### 3.1. Patient and Disease Characteristics

In total, 48 patients with 89 target tumors underwent iBT including 60 completed therapy cycles, while 47 patients with 70 target tumors received combined cTACE and iBT, including 51 completed therapy cycles. The mean age was 70.6 ± 9.3 and 70.3 ± 9.5 years in patients following iBT and cTACE/iBT (*p* = 0.84), and 79.2% vs. 76.6% were male (*p* = 0.81), respectively. Patients undergoing iBT had 1.4 ± 0.7 lesions (uni- vs. multifocal: 41 vs. 19) at baseline with a mean index lesion diameter of 26.5 ± 10.6 mm, and patients treated with cTACE/iBT had 1.5 ± 0.9 lesions (uni- vs. multifocal: 38 vs. 13, *p* = 0.53) with a mean index lesion diameter of 44.3 ± 20.4 mm (*p* < 0.01), respectively. Patients, tumor, and disease characteristics and laboratory values are summarized in Table 1.

### 3.2. Tumor Response

Tumor response assessments via follow-up imaging were feasible for 99 completed treatment cycles (89.2%) two months post-LRT (median [IQR]: 2.3 [2.0, 2.8] months) as well as for 92 completed treatment cycles (82.9%) five months post-LRT (median [IQR]: 5.3 [4.8, 6.2] months) mainly to due deviating from imaging protocols or events like OLT or death during follow-up. The survival measure times for non-responders vs. responders following both iBT and cTACE/iBT, respectively, as well as the log-rank test’s *p*-values are reported in detail in Supplementary Table S1 (iBT), Supplementary Table S2 (cTACE/iBT), and Table 2 (log-rank test’s *p*-values).

The size-based criteria identified significantly less responders than non-responders at two months post-iBT (RECIST: 19.2% vs. 80.8%, *p* < 0.01, and WHO: 21.2% vs. 78.8%, *p* < 0.01) and at five months post-iBT (RECIST: 20.4% vs. 79.6%, *p* = 0.01, WHO: 30.6% vs. 69.4%, *p* = 0.01) as well as at two months post-cTACE/iBT (RECIST: 6.4% vs. 93.6%, and WHO 10.6% vs. 89.4%, *p* < 0.01) and at five months post-cTACE/iBT (RECIST: 13.9% vs. 86.1%, and WHO: 16.3% vs. 83.7%, *p* < 0.01).

**Figure 1:**
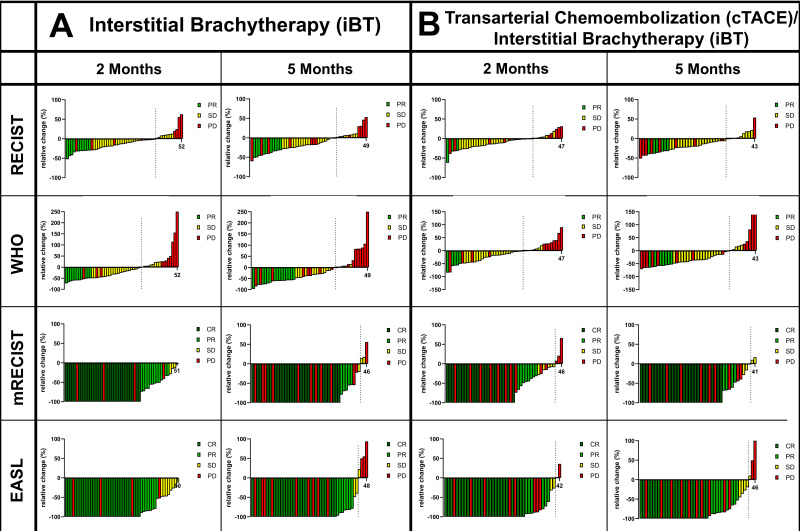
Waterfall plots illustrating the relative tumoral change within completed treatment cycles according to the respective response criteria of RECIST, WHO, mRECIST, and EASL at two and five months of follow-up for (**A**) patients following interstitial brachytherapy (iBT) and (**B**) following conventional transarterial chemoembolization (cTACE)/iBT. Response categories include complete response (CR), partial response (PR), stable disease (SD), and progressive disease (PD).

In contrast, the enhancement-based criteria identified more responders than non-responders at two months post-iBT (mRECIST: 84.3% vs. 15.7%, *p* < 0.01, EASL: 76.5% vs. 23.5%, *p* = 0.01) and at five months post-iBT (mRECIST: 66.7% vs. 33.3%, *p* = 0.02, EASL: 70.8% vs. 28.8%, *p* = 0.01) as well as at two months post-cTACE/iBT for mRECIST (61.7% vs. 38.3%, *p* = 0.03), but not for the EASL at two months (53.2% vs. 46.8%, *p* = 0.67) or at five months post-cTACE/iBT (mRECIST: 66.7% vs. 33.3%, *p* = 0.01, EASL: 71.4% vs. 38.6%, *p* < 0.01, Figure 1).

**Figure 2:**
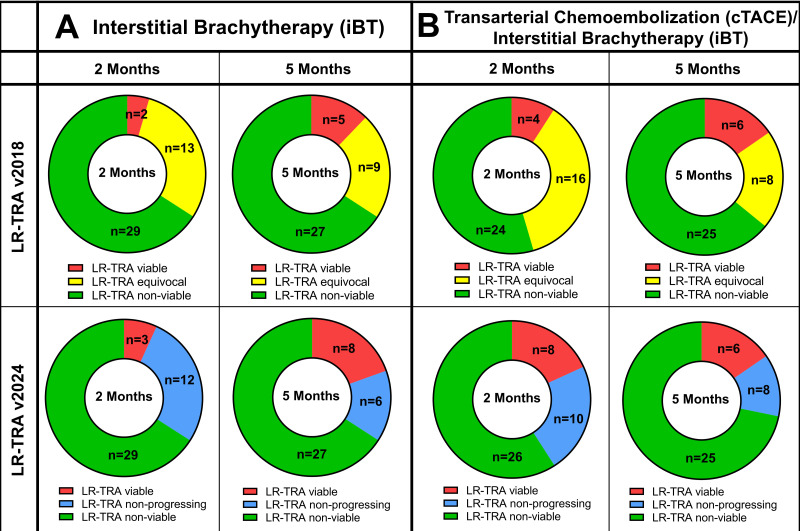
Donut plots illustrate the distribution of tumor response according to the response categories of LIRADS Treatment Response Algorithm (LR-TRA) v2018 and v2024 radiation core at two and five months of follow-up for (**A**) patients following interstitial brachytherapy (iBT) and (**B**) following conventional transarterial chemoembolization (cTACE)/iBT.

The LR-TRA v2018 and v2024 radiation core identified more responders than non-responders at two months post-iBT (responders vs. non-responders, LR-TRA v2018: 67.3% vs. 32.7%, *p* = 0.02, LR-TRA v2024: 74.6% vs. 25.4%, *p* < 0.01) and at five months post-iBT (LR-TRA v2018: 71.4% vs. 28.6%, *p* = 0.01, LR-TRA v2024: 65.9% vs. 34.1%, *p* = 0.03) as well as at two months post-cTACE/iBT (LR-TRA v2018: 68.7% vs. 31.3%, *p* = 0.02, LR-TRA v2024: 71.0% vs. 29.0%, *p* < 0.01) and at five months post-cTACE/iBT for the LR-TRA v2024 (71.8% vs. 28.2%, *p* < 0.01) but not for the LR-TRA v2018 (64.1% vs. 35.9%, *p* = 0.06; Figure 2).

### 3.3. Predicting OS, PFS, and TTP Regarding Response Criteria

The median OS and PFS did not differ between patients following iBT or cTACE/iBT (iBT vs. cTACE/iBT, median OS [IQR]: 26.3 [14.4, 45.3] months vs. 23.3 [13.8, 47.6] months, *p* = 0.71, and median PFS [IQR]: 9.1 [4.7, 15.4] months vs. 7.6 [2.6, 19.4] months, *p* = 0.39). However, TTP was significantly longer in the iBT group compared to the cTACE/iBT group (median [IQR]: 13.0 [5.8, 43.7] months vs. 9.2 [4.7, 19.6] months, *p* = 0.04) (Supplementary Figure S1).

iBT patients: In patients undergoing iBT, stratification according to the size-base criteria as early as two months post-iBT did not yield significant differences in patient survival. However, at five months post-iBT, the responders showed prolonged PFS_LTP_ times compared to non-responders by RECIST (responders vs. non-responders, median [IQR]: 11.0 [4.7, 18.8] months vs. 0.8 [0.4, 11.1] months, *p* < 0.01).

**Figure 3:**
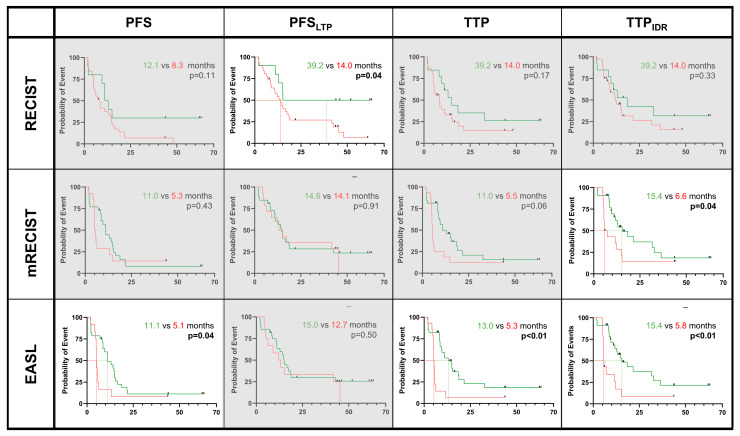
Survival measures in patients following interstitial brachytherapy (iBT) alone. Green lines indicate responders vs. non-responders (red lines) according to the respective response criteria at five months of follow-up. Significant results were found for RECIST, mRECIST, and EASL and are highlighted next to grayed-out boxes. Survival times are reported in Supplementary Table S1.

As for the enhancement-based criteria that were assessed as early as two months of follow-up, no significant differences in patients’ survival were observed. However, at five months post-iBT, responders showed a prolonged TTP_IDR_ subtype according to mRECIST (median [IQR]: 15.4 [9.4, 32.7] months vs. 6.6 [5.4, 14.8] months, *p* = 0.05), while responders showed a prolonged PFS time according to the EASL (median [IQR]: 11.1 [7.0, 16.50] months vs. 5.1 [4.7, 5.8] months, *p* = 0.04), TTP (median [IQR]: 13.0 [8.3, 21.6] months vs. 5.3 [4.9, 5.9] months *p* < 0.01) and TTP_IDR_ subtype (median [IQR]: 15.4 [9.4, 36.4] months vs. 5.8 [5.4, 12.2] months, *p* < 0.01) compared to non-responders (Figure 3).

The LR-TRA v2018 criteria and v2024 radiation core revealed no significant trends in patients’ survival at any follow-up time point post-iBT.

cTACE/iBT patients: In patients following combined cTACE and iBT, the size-base criteria did not show significant predictions for any survival measure that was assessed two or five months post-cTACE/iBT.

However, regarding the enhancement-based criteria, mRECIST significantly separated the survival curves of responders and non-responders at two months post-cTACE/iBT for the PFS and PFS_IDR_ subtypes and for the TTP and TTP_IDR_ subtypes and at five months post-cTACE/iBT for the TTP and TTP_IDR_ subtypes, while responders showed better PFS and PFS_IDR_ subtypes according to the EASL as well as the TTP and TTP_IDR_ subtypes at both two and five months post-cTACE/iBT.

**Figure 4:**
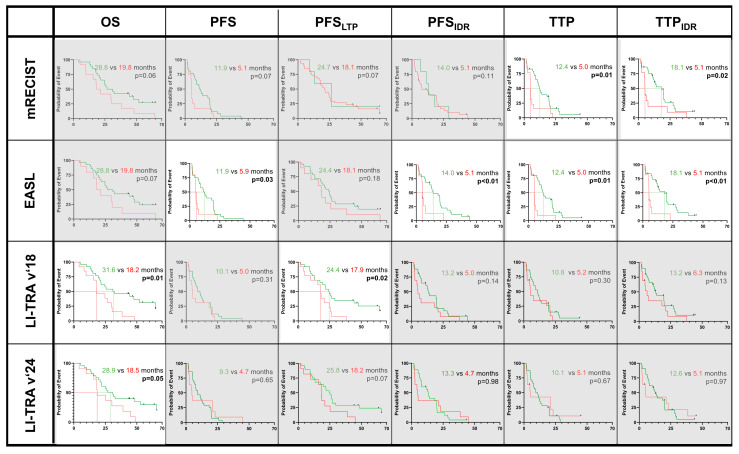
Survival measures in patients following conventional transarterial chemoembolization (cTACE) prior to interstitial brachytherapy (iBT). Green lines indicate responders vs. non-responders (red lines) according to the respective response criteria at five months of follow-up. Significant results were found for mRECIST, EASL, and both LI-TRA criteria and are highlighted next to grayed-out boxes. Survival times are reported in Supplementary Table S2.

Notably, responders according to the EASL predicted prolonged PFS assessed as early as after two months (median [IQR]: 11.0 [5.2, 19.6] months vs. 2.3 [1.9, 5.2] months, *p* < 0.01) and after five months post-cTACE/iBT (median [IQR]: 11.9 [4.2, 19.6] months vs. 5.1 [4.7, 6.1] months, *p* = 0.03), PFS_IDR_ after two months (median [IQR]: 13.2 [7.5, 19.9] months vs. 2.3 [1.9, 6.3] months, *p* < 0.01) and after five months post-cTACE/iBT (median [IQR]: 14.0 [6.9, 20.9] months vs. 5.1 [4.7, 7.5] months, *p* = 0.01), TTP after two months (median [IQR]: 11.9 [6.1, 19.8] months vs. 2.4 [1.9, 5.2] months, *p* < 0.01) and after five months post-cTACE/iBT (median [IQR]: 12.4 [7.6, 19.9] months vs. 5.0 [4.7, 6.1] months, *p* < 0.01), and TTP_IDR_ after two months (median [IQR]: 18.1 [7.6, 28.2] months vs. 2.4 [1.9, 6.3] months, *p* < 0.01) and after five months post-cTACE/iBT (median [IQR]: 18.1 [7.9, 27.4] months vs. 5.1 [4.7, 6.1] months, *p* < 0.01), respectively (Figure 4).

Additionally, the LR-TRA criteria measured as late as five months post-cTACE/iBT revealed prolonged OS for responders (LR-TRA v2018: median OS [IQR]: 31.6 [19.4, 64.6] months vs. 18.2 [15.6, 29.7] months, *p* < 0.01 and LR-TRA v2024: median OS [IQR]: 29.0 [17.5, 65.7] months vs. 18.5 [15.8, 38.9] months, *p* = 0.05) as well as prolonged PFS_LTP_ (LR-TRA v2018: median PFS_LTP_ [IQR]: 24.4 [12.9, 64.6] months vs. 17.9 [9.7, 24.7] months, *p* = 0.05, but not for the LR-TRA v2024 radiation core: median PFS_LTP_ [IQR]: 24.8 [11.2, 48.4] months vs. 18.2 [9.8, 21.0] months, *p* = 0.07). However, LR-TRA did not reveal any significant trends in patient survival stratified by response criteria at two months post-cTACE/iBT.

### 3.4. Confounders of OS, PFS, and TTP

The univariate Cox proportional hazard models revealed age (*p* = 0.07), sum of lesion diameter (*p* = 0.02), BCLC stage C (*p* = 0.09), and ALBI score (*p* < 0.01) as significant predictors of OS. Moreover, sum of lesion diameter (*p* = 0.08) and ALBI score (*p*<0.01) were confirmed as such in a subsequent multivariate analysis. Regarding PFS and TTP, only the ALBI score was significantly associated (*p* = 0.09) with PFS and BCLC stage C (*p* = 0.09) with TTP (Supplementary Table S3). Hereby, a consecutive multivariate analysis was obsolete.

## 4. Discussion

This study revealed the superiority of enhancement-based response criteria (mRECIST, EASL, and LR-TRA) over size-based criteria (RECIST 1.1 and WHO) in predicting outcomes in patients with HCC following both iBT and cTACE/iBT. Specifically, responders identified by mRECIST and the EASL showed significantly better PFS and TTP. The overall favorable response following iBT was measurable later than that following the combination of iBT with prior cTACE. While the LR-TRA did not predict tumor progression, it identified responders with better OS and PFS related to local tumor progression (PFS_LTP_) compared to non-responders.

Size-based criteria, particularly RECIST 1.1, remain widely used for assessing treatment response in solid tumors due to their simplicity and established prognostic potential [20,21]. However, RECIST was originally designed to evaluate morphological changes in tumor size following conventional chemotherapies. Thus, it may underestimate treatment responses induced by LRT, which often result in necrosis and fibrosis without immediate tumor shrinkage. In contrast, mRECIST and EASL criteria, which consider tumor devascularization and necrosis as response patterns, are more suitable for evaluating LRT, especially embolotherapies [22,23]. Accordingly, this study demonstrated higher numbers of responders according to mRECIST and the EASL and poorer PFS and TTP values for non-responders following both iBT and combined cTACE/iBT. Hence, these findings suggest that besides treatment modality, the tumor’s imaging appearance and characteristics (e.g., hypervascularization) should be considered when defining response patterns.

While both mRECIST and the EASL were equally applicable and prognostic for patients post-cTACE/iBT, the EASL proved more reliable post-iBT, highlighting the superior prognostic value of bidimensional measurements in this context. Unidimensional assessments may not adequately assess atypical necrosis patterns or may overestimate post-therapeutic perifocal hyperemia as viable tumor tissue [24].

Unlike thermal ablation techniques, iBT induces gradual necrosis without immediate tumor shrinkage, which explains the lower initial numbers of responders given by the size-based criteria. Additionally, the study revealed that more responders were detected at five months of follow-up compared to eight weeks using enhancement-based tools, suggesting that follow-up visits should not be scheduled too early to prevent unnecessary additional therapies and to reduce clinical healthcare costs due to over-surveillance.

Despite their prognostic value, size- and enhancement-based uni- and bidimensional measures are criticized for their simplicity, potentially failing to capture the complexity of tumor heterogeneity and resilience mechanisms, particularly in heterogeneous tumors like HCC that develop in cirrhotic livers [25]. Advanced tools such as three-dimensional (3D) quantitative (q)EASL promise more accurate tumor response assessments, but their implementation in clinical practice is hindered by their labor-intensive complexity and the difficulties encountered in terms of software distribution and user friendliness [24,26].

In comparison, LR-TRA criteria were developed as a semantic feature-based algorithm to facilitate treatment response assessment in HCC lesions [9,12]. The updated LR-TRA v2024 catalog includes two separate algorithms for non-radiation and radiation-based therapies [10] replacing the nowadays obsolete v2018 catalog. However, in this study, both the old and new LR-TRA radiation core algorithms were included to evaluate their advantages. This study found the LR-TRA to have a prognostic value for OS (both v2018 and v2024) and for PFS related to local tumor progression (PFS_LTP_ for LR-TRA v2018 only), possibly due to its comprehensive approach considering major criteria like size and enhancement changes, as well as the recently added criteria of T2-weighted and diffusion-weighted imaging, indicating tumor viability or necrosis patterns. However, in this study, the LR-TRA v2024 radiation core did not show superiority compared to the standard LR-TRA v2018 core at predicting survival measures post-LRT. The LR-TRA may in fact better reflect local post-therapeutic alterations indicating local tumor recurrence and poorer patient survival by including various imaging features; however, these alterations are heterogeneous and may vary among patients since they might be affected by the underlying liver disease and tumor subtype. Moreover, higher inter-reader variability could limit the LR-TRA’s prognostic efficacy [27]. Therefore, commonly cited radiomics could expand the promising approach of incorporating ancillary imaging findings into diagnostic algorithms that reveal mutual image information and may better reflect tumor heterogeneity at baseline as well as in follow-up imaging post-LRT. This could possibly help to design personal treatment strategies and support early follow-up prognostics [28]. Similarly, mutual tumor burden biomarkers are currently being investigated (e.g., circulating tumor DNA (ctDNA)), showing promise in predicting early tumor responses for various cancer types following surgical, local ablative, and systemic treatments [29]. Overall, categorizing treatment response following iBT remains challenging since it is classically considered a radiation-based modality and could potentially be evaluated similarly to non-radiation-based RFA and MWA due to its ablative nature.

Given its retrospective single-center design, this study has some limitations. A limited number of patients were included in each treatment group. While patients in both groups shared similar age and gender distributions, they differed in terms of tumor size, lesion count, and some baseline lab values. However, the comparison of both groups was not the focus of this study, and the results from both treatments were evaluated separately. Moreover, they did not differ regarding OS or PFS, but patients receiving monotherapy via iBT alone had better TTP compared to patients with combined cTACE/iBT since the treatments were allocated by the MTB depending on the disease stage and tumor extent. Usually, patients that were suitable for cTACE/iBT combination therapy had larger lesions and therefore a probable more severe underlying liver disease, which is favorable for a consecutive event of IDR. Although underlying liver disease and tumor characteristics are known prognostic markers for the outcome of patients with HCC, this study focused on the prognostic value of the tested response criteria to support their potential for clinical implementation in a real-life setting. The reproducibility of the study’s findings may rely on the MRI scanner and protocol as well as the contrast agent. Lastly, the follow-up imaging time points were defined by the routine clinical schedule of our tertiary care center. Future studies are needed to identify the exact onset of measurable therapy-induced alterations following LRT.

## 5. Conclusions

In conclusion, the enhancement-based criteria proved to be more reliable tools compared to the size-based criteria in determining treatment response in patients with HCC following ablation via iBT alone or iBT combined with prior cTACE. While prior embolotherapy resulted in measurable tumor necrosis as early as eight weeks post-LRT, response to iBT mostly became measurable after five months of follow-up. These findings highlight the importance of considering tumor biology and imaging characteristics in response assessments. Efficient monitoring strategies should be tailored to the treatment modality and the timing to obtain a measurable response to avoid unnecessary additional treatments.

## Figures and Tables

**Table 1:** Baseline patient, tumor, and disease characteristics.

Demographics	iBT Patients	cTACE/iBT Patients	*p*-Value
**Patient characteristics**			
Number of patients	48	47	-
Age, *mean ± SD*	70.6 ± 9.3 years	70.3 ± 9.5 years	0.84
Male:Female, *n*	38:10	36:11	0.81
**Tumor characteristics**			
Lesions, *n*	89	70	-
Sum of lesion diameter, *mean ± SD*	26.5 ± 10.6 mm	44.3 ± 20.4 mm	**<0.01**
Unifocal:Multifocal, *n*	41:19	38:13	0.53
Completed treatment cycles, *n*	60	51	-
HCC diagnosis, *n (biopsy* vs. *imaging)*	43:17	24:27	-
HCC grading, *n (%)*			
GX	28 (65.1%)	6 (25.0%)	-
G1	1 (2.3%)	2 (8.3%)	-
G2	7 (16.3%)	13 (54.2%)	-
G3	7 (16.3%)	3 (12.5%)	-
**Disease characteristics**			
Cirrhosis, *n (%)*	41 (85.4%)	43 (91.5%)	0.52
Etiology of cirrhosis, *n (%)*			
Viral hepatitis (Hepatitis B/C)	12 (29.3%)	11 (23.2%)	0.98
Alcoholic steatohepatitis	11 (26.8%)	18 (41.9%)	0.17
Metabolic-dysfunction associated steatohepatitis	13 (31.7%)	14 (32.6%)	0.99
Others *	5 (12.2%)	0 (0.0%)	**0.02**
Child Pugh class, *n (%)*			
A	34 (82.9%)	41 (97.7%)	0.09
B	7 (17.1%)	2 (2.3%)	0.09
Barcelona Clinic Liver Cancer stage, *n (%)*			
A	41 (85.4%)	22 (46.8%)	**<0.01**
B	6 (12.5%)	17 (36.2%)	**0.01**
C	1 (2.1%)	8 (17.0%)	**0.02**
**Laboratory values of liver function**, *mean ± SD or median [IQR]*			
Alpha-fetoprotein (AFP) (U/mL)	8.0 [5.4, 46.2]	13.1 [5.2, 69.8]	0.46
Albumine (g/L)	39.7 [35.3, 42.9]	39.0 [37.3, 40.5]	0.96
Bilirubin (mg/dL)	0.6 [0.4, 1.1]	0.7 [0.5, 1.1]	0.23
ALBI dcore	−2.56 ± 0.37	−2.56 ± 0.60	0.96
AST/ALT tatio	1.3 [1.0, 1.5]	1.2 [1.1, 1.5]	0.67
γ-GT (U/L)	119 [56, 218]	152 [105, 240]	0.24
AP (U/L)	3.4 [0.9, 5.6]	4.4 [2.4, 6.7]	0.73

iBT interstitial high-dose-rate brachytherapy, cTACE conventional transarterial chemoembolization, ALT alanine aminotransferase, AST aspartate aminotransferase, γ-GT gamma-glutamyl transferase. Bold *p*-values indicate statistical significance (Fisher’s exact test, unpaired *t*-test, and Mann–Whitney U test, respectively). * Other etiologies of cirrhosis comprise hereditary diseases like PSC or hemochromatosis and medication- or toxin-related causes.

**Table 2:** Log-rank test’s *p*-values for OS, PFS, TTP, and PFS/TTP subtypes according to response assessment criteria for patients at two and five months following iBT and iBT/cTACE.

		iBT							cTACE/iBT						
	Criterion	OS	PFS	PFS	PFS	TTP	TTP	TTP	OS	PFS	PFS	PFS	TTP	TTP	TTP
				LTP	IDR		LTP	IDR			LTP	IDR		LTP	IDR
**2 Months**	RECIST	0.52	0.96	0.80	0.79	0.83	0.78	0.77	0.83	0.86	0.72	0.64	0.56	0.53	0.54
	WHO	0.24	0.18	0.26	0.08	0.63	0.61	0.28	0.21	0.13	0.31	0.09	0.93	0.99	0.67
	mRECIST	0.52	0.15	0.33	0.98	0.65	0.20	0.32	0.99	**<0.01**	0.9	**<0.01**	**<0.01**	0.77	**<0.01**
	EASL	0.91	0.80	0.82	0.59	0.19	0.24	0.08	0.81	**<0.01**	0.86	**<0.01**	**<0.01**	0.72	**<0.01**
	LI-TRA v2018	0.87	0.67	0.48	0.50	0.93	0.34	0.08	0.13	0.42	0.24	0.88	0.60	0.31	0.95
	LI-TRA v2024	0.61	0.98	0.32	0.73	0.98	0.22	0.93	0.06	0.81	**0.01**	0.29	0.27	0.21	0.18
**5 Months**	RECIST	0.12	0.11	**0.04**	0.13	0.17	0.26	0.33	0.17	0.83	0.65	0.91	0.80	0.44	0.62
	WHO	0.86	0.43	0.14	0.91	0.75	0.17	0.72	0.55	0.87	0.75	0.71	0.61	0.64	0.92
	mRECIST	0.90	0.43	0.91	0.50	0.06	0.61	**0.04**	0.06	0.07	0.07	0.11	**0.01**	0.62	**0.02**
	EASL	0.82	**0.04**	0.50	0.08	**<0.01**	0.62	**<0.01**	0.07	**0.03**	0.18	**<0.01**	**0.01**	0.93	**<0.01**
	LI-TRA v2018	0.40	0.72	0.92	0.68	0.57	0.26	0.57	**0.01**	0.31	**0.02**	0.14	0.3	0.18	0.13
	LI-TRA v2024	0.65	0.91	0.98	0.76	0.77	0.31	0.91	**0.05**	0.65	0.07	0.98	0.67	0.64	0.97

Legend: iBT interstitial high-dose-rate brachytherapy, cTACE conventional transarterial chemoembolization, OS overall survival, PFS progression-free survival, TTP time to progression, LTP local tumor progression, IDR intrahepatic distant recurrence. *p*-values indicate differences in the log-rank test between responders and non-responders according to the respective response criteria. The red-green color gradient indicates the strength of the *p*-value. Significant *p*-values are bold printed. Survival times are reported in Supplementary Tables S1 and S2.

## Data Availability

Additional data are available to readers upon reasonable request to the corresponding author.

## Supplementary Materials

The following supporting information can be downloaded at: https://www.mdpi.com/article/10.3390/cancers17081275/s1, Table S1: Survival measures comparing patients stratified as non-responders vs. responders following interstitial brachytherapy (iBT); Table S2: Survival measures comparing patients stratified as non-responders vs. responders following conventional chemoembolization (cTACE)/interstitial brachytherapy (iBT); Table S3: Uni- and multivariate cox proportional hazard model results; Figure S1: Overall survival (OS), progression-free survival (PFS) and time to progression (TTP) in patients following interstitial brachytherapy (iBT) alone or with a combined 38 prior conventional transarterial chemoembolization (cTACE/iBT).

## Abbreviations

The following abbreviations are used in this manuscript:

ALBI score	Albumin–bilirubin score
iBT	Interstitial (high-dose-rate) brachytherapy
CT	Computer tomography
EASL	European Association for the Study of the Liver
ESMO	European Society of Medical Oncology
HCC	Hepatocellular carcinoma
IDR	Intrahepatic distant recurrence
IQR	Interquartile range
MRI	Magnetic resonance imaging
MTB	Multidisciplinary Tumor Board
MWA	Microwave ablation
LI-RADS	Liver Imaging and Reporting and Data System
LR-TRA	LIRADS Treatment Response Algorithm
LRT	Locoregional therapy
LTP	Local tumor progression
OS	Overall survival
PFS	Progression-free survival
(m)RECIST	(Modified) Response Evaluation Criteria in Solid Tumors
RFA	Radiofrequency ablation
SD	Standard deviation
cTACE	Conventional transarterial chemoembolization
TTP	Time to progression
WHO	World Health Organization
