# Supplementary material for: Comparing the Prognostic Value of Quantitative Response Assessment Tools and LIRADS Treatment Response Algorithm in Patients with Hepatocellular Carcinoma Following Interstitial High-Dose-Rate Brachytherapy and Conventional Transarterial Chemoembolization
Source: Cancers (Basel). 2025 Apr 9;17(8):1275. doi: 10.3390/cancers17081275 (PMC12025668; doi:10.3390/cancers17081275)
Supplement: Supplementary file 1 [file cancers-17-01275-s001.zip › cancers-3506207-supplementary.pdf]

Supplementary Material

Comparing the Prognostic Value of Quantitative Response Assessment Tools and LIRADS Treatment Response Algorithm in Patients with Hepatocellular Carcinoma Following Interstitial High Dose Rate Brachytherapy and Conventional Transarterial Chemoembolization

Robin Schmidt [1,2], Christopher Rueger [1], Han Xu [1], Yubei He [1,2], Emine Yaren Yilmaz [1,2], Luisa Heidemann [1,2], Ornela Sulejmani [1,2], Yu Liu [1,2], Lasse Noack [1], Friederike Hesse [1,3], Richard Ruppel [1], Sara Abosabie [1], Charlie Alexander Hamm [1,3], Tobias Penzkofer [1], Bernhard Gebauer [1], and Lynn Jeanette Savic [1,2,3]*

[1] Charité – Universitätsmedizin Berlin, corporate member of Freie Universität Berlin and Humboldt-Universität zu Berlin, Department of Radiology, Augustenburger Platz 1, 13353 Berlin, Germany
[2] Experimental Clinical Research Center (ECRC) at Charité - Universitätsmedizin Berlin and Max-Delbrück-Centrum für molekulare Medizin (MDC), Robert-Rössle-Straße 10, 13125 Berlin, Germany
[3] Berlin Institute of Health at Charité – Universitätsmedizin Berlin, Charitéplatz 1, 10117 Berlin, Germany
* Correspondence: PD Dr. med. Lynn Jeanette Savic lynn-jeanette.savic@charite.de

Supplementary Material

Supplement Table S1. Survival measures comparing patients stratified as non-responders vs. responders following interstitial brachytherapy (iBT).

iBT		Survival measures in months for non-responders vs. responders, median [IQR]						
Time	Criterion	OS	PFS	PFS_{LTP}	PFS_{IDR}	TTP	TTP_{LTP}	TTP_{IDR}
2 Months	RECIST	22.3 [14.2,46.9] vs. 26.4 [15.4,37.4]	9.6 [4.9,15.7] vs. 6.4 [5.1,7.0]	14.6 [8.3,48.1] vs. 11.1 [5.1,46.0]	11.6 [5.5,16.5] vs. 7.0 [5.8,7.0]	9.6 [5.4,32.7] vs. 9.4 [5.1,10.3]	59.8 [14.6,n/d] vs. n/d [n/d,n/d]	12.2 [8.2,44.8] vs. 13.0 [5.5,10.3]
	WHO	36.3 [15.1,63.8] vs. 15.1 [12.2,41.7]	10.9 [5.1,16.5] vs. 5.4 [4.9,15.4]	14.6 [8.3,48.1] vs. 13.7 [11.1,41.7]	13.0 [5.8,15.4] vs. 9.2 [8.3,22.7]	9.6 [5.1,21.6] vs. 8.2 [4.9,18.4]	n/d [9.1,n/d] vs. n/d [35.2,n/d]	13.0 [8.3,39.7] vs. 12.2 [5.8,18.4]
	mRECIST	36.3 [12.8,55.6] vs. 26.3 [15.0,45.2]	10.9 [2.1,39.7] vs. 9.1 [5.0,15.0]	13.1 [10.7,55.6] vs. 14.6 [7.4,41.7]	10.9 [2.1,39.7] vs. 12.7 [7.3,15.7]	6.5 [2.1,39.7] vs. 9.4 [5.1,18.8]	n/d [n/d,n/d] vs. n/d [9.1,n/d]	6.5 [2.1,39.7] vs. 14.8 [8.3,31.6]

	**EASL**	25.8 [12.8,55.6] vs. 26.3 [15.0,45.3]	7.4 [2.1,13.1] vs. 9.1 [5.1,15.4]	13.0 [10.7,55.6] vs. 14.6 [8.6,42.7]	9.6 [2.1,11.6] vs. 13.0 [8.3,16.5]	6.7 [11.6,2.1] vs. 11.0 [5.4,18.8]	n/d [n/d,n/d] vs. n/d [9.1,n/d]	8.2 [2.1,11.6] vs. 15.3 [8.6,32.7]
	**LI-TRA v2018**	15.2 [12.8,61.1] vs. 29.6 [15.7,45.2]	16.2 [13.1,63.1] vs. 8.6 [4.9,14.6]	13.1 [7.4,61.1] vs. 15.0 [8.6,42.7]	11.6 [7.0,14.6] vs. 13.0 [7.3,22.7]	11.0 [5.5,21.6] vs. 8.6 [4.9,18.8]	n/d [9.1,n/d] vs. n/d [35.2,n/d]	11.8 [9.6,21.6] vs. 13.0 [5.8,36.4]
	**LI-TRA v2024**	26.4 [13.3,62.1] vs. 30.1 [15.9,45.9]	11.1 [5.6,14.9] vs. 8.7 [4.7,15.9]	14.1 [11.1,62.1] vs. 15.2 [8.4,42.4]	11.8 [2.4,18.4] vs. 13.3 [5.1,20.1]	11.1 [5.6,21.9] vs. 9.3 [4.9,19.1]	n/d [n/d,n/d] vs. n/d [8.7,n/d]	11.9 [8.4,40.4] vs. 13.2 [5.9,33.2]
**5 Months**	**RECIST**	21.8 [14.6,45.2] vs. 49.8 [15.2,63.8]	8.3 [4.7,14.8] vs. 12.1 [9.6,63.2]	14.0 [7.0,41.7] vs. 39.2 [12.8,63.2]	12.2 [9.8,14.9] vs. 12.1 [5.9,23.1]	8.3 [5.1,21.6] vs. 15.0 [9.6,63.2]	n/d [8.3,n/d] vs. n/d [8.6,n/d]	12.2 [7.3,31.6] vs. 18.4 [9.6, n/d]
	**WHO**	33.2 [15.0,45.3] vs. 22.9 [15.2,62.3]	11.0 [8.6,21.9] vs. 8.3 [4.9,15.4]	14.0 [8.3,42.7] vs. 16.9 [12.8,62.3]	14.0 [7.3,21.6] vs. 10.3 [8.3,62.3]	8.3 [5.0,21.6] vs. 10.9 [8.3,18.4]	n/d [8.3,n/d] vs. n/d [35.2,n/d]	14.8 [5.8,32.7] vs. 10.9 [8.3, 18.4]
	**mRECIST**	31.6 [15.0,45.2] vs. 23.8 [14.6,45.3]	5.3 [4.9,13.1] vs. 11.0 [7.0,15.4]	14.1 [6.3,45.2] vs. 14.6 [8.3,42.7]	7.3 [5.5,14.8] vs. 13.0 [8.6,21.6]	5.5 [5.0,11.6] vs. 11.0 [8.3,21.6]	n/d [6.3,n/d] vs. n/d [15.0,n/d]	6.6 [5.5,14.8] vs. 15.4 [9.4, 32.7]
	**EASL**	28.9 [15.0,45.2] vs. 26.3 [14.6,63.2]	5.1 [2.1,4.9] vs. 11.1 [8.3,16.5]	12.7 [6.3,45.2] vs. 15.0 [8.6,63.2]	6.6 [5.5,12.2] vs. 14.0 [8.6,2.7]	5.3 [4.9,5.8] vs. 13.0 [8.3,21.6]	n/d [5.1,n/d] vs. n/d [15.0,n/d]	5.8 [5.4,12.2] vs. 15.4 [9.4,36.4]
	**LI-TRA v2018**	36.6 [15.0,63.8] vs. 22.9 [14.6,45.2]	8.3 [2.6,14.8] vs. 11.0 [5.1,15.4]	13.0 [5.1,61.1] vs. 15.1 [10.7,45.2]	11.6 [7.0,22.3] vs. 14.0 [7.3,22.7]	9.6 [2.6,14.8] vs. 9.4 [5.5,18.8]	n/d [4.7,n/d] vs. n/d [15.0,n/d]	11.8 [9.6,21.6] vs. 15.3 [7.3,36.4]
	**LI-TRA v2024**	31.8 [14.6,62.1] vs.	10.4 [4.7,15.0] vs.	13.2 [7.1,62.1] vs.	11.8 [7.1,15.0] vs.	11.7 [4.7,15.0] vs.	n/d [5.2,n/d] vs.	12.4 [7.4,36.9] vs.

		27.7 [15.3,64.9]	8.7 [5.1,15.6]	15.4 [8.7,45.9]	13.2 [7.4,23.1]	8.7 [5.5,19.1]	n/d [15.2,n/d]	13.2 [9.8,21.9]

OS overall survival, PFS progression-free survival, TTP time-to-progression, LTP local tumor progression subtype, IDR intrahepatic distant recurrence subtype, RECIST Response Evaluation Criteria in Solid Tumors v1.1, mRECIST modified RECIST, WHO World Health Organization, EASL European Study of the Liver, LI-TRA Liver Imaging and Reporting Data System (LIRADS) Treatment Response Algorithm, n/d non-defined. Data is depicted as median and interquartile range (IQR).

Supplement Table S2. Survival measures comparing patients stratified as non-responders vs. responders following conventional chemoembolization (cTACE)/interstitial brachytherapy (iBT).

cTACE/iBT		Survival measures in months for Non-Responders vs. Responders, median [IQR]						
**Time**	**Criterion**	**OS**	**PFS**	**PFS$_{LTP}$**	**PFS$_{IDR}$**	**TTP**	**TTP$_{LTP}$**	**TTP$_{IDR}$**
**2 Months**	**RECIST**	24.0 [15.1,48.0] vs. 11.0 [8.1,13.8]	7.6 [2.5,19.4] vs. 9.1 [5.2,12.9]	20.1 [9.2,29.1] vs. 9.1 [5.2,12.9]	7.9 [2.6,19.8] vs. 9.6 [6.3,12.9]	7.9 [2.6,19.6] vs. 12.9 [5.2,27.4]	n/d [25.6,n/d] vs. 12.9 [5.2,n/d]	11.9 [4.7,19.9] vs. 27.4 [6.3,27.4]
	**WHO**	24.7 [6.0,48.0] vs. 6.9 [5.8,13.0]	8.4 [2.6,19.6] vs. 4.7 [4.2,7.9]	21.1 [9.9,38.3] vs. 7.5 [5.2,13.0]	11.9 [3.2,19.8] vs. 6.3 [4.2,7.9]	9.1 [2.6,19.6] vs. 7.9 [5.2,27.4]	n/d [20.7,n/d] vs. n/d [26.4,n/d]	12.0 [4.7,19.9] vs. 7.9 [6.3,27.4]
	**mRECIST**	18.2 [8.7,48.0] vs. 23.3 [15.1,47.6]	2.3 [1.9,5.2] vs. 11.3 [5.2,19.6]	17.9 [5.3,38.3] vs. 21.4 [9.9,26.6]	2.3 [1.9,6.3] vs. 13.2 [7.6,19.9]	2.4 [2.1,7.5] vs. 11.9 [6.1,19.8]	n/d [24.1,n/d] vs. n/d [n/d,n/d]	2.4 [2.1,7.5] vs. 18.1 [7.9,27.4]
	**EASL**	18.2 [8.7,51.9] vs. 23.3 [15.1,44.3]	2.3 [1.9,5.2] vs. 11.0 [5.2,19.6]	17.9 [5.3,38.3] vs. 21.4 [9.9,26.4]	2.3 [1.9,6.3] vs. 13.2 [7.5,19.9]	2.4 [2.1,11.3] vs. 11.9 [6.1,19.8]	n/d [20.7,n/d] vs. n/d [24.1,n/d]	2.4 [2.1,11.3] vs. 18.1 [7.6,27.4]
	**LI-TRA v2018**	22.0 [15.6,38.8] vs. 23.1 [13.8,64.6]	6.4 [2.5,20.7] vs. 7.8 [3.2,19.4]	18.5 [8.7,26.4] vs. 20.4 [9.7,47.6]	6.9 [2.5,20.9] vs. 12.4 [5.0,19.6]	7.5 [2.5,20.7] vs. 9.1 [4.8,19.6]	26.4 [20.7,n/d] vs. n/d [24.1,n/d]	7.5 [2.5,20.9] vs. 12.4 [5.1,19.9]
	**LI-TRA v2024**	21.1 [8.8,30.9] vs. 23.5 [14.0,65.7]	5.2 [2.3,18.3] vs. 8.6 [3.2,19.7]	18.4 [5.9,26.0] vs. 20.8 [9.8,65.7]	5.8 [2.4,18.4] vs. 13.1 [5.1,20.1]	11.2 [4.7,21.0] vs. 7.7 [2.3,19.9]	26.9 [21.0,n/d] vs. n/d [24.5,n/d]	12.6 [4.7,23.0] vs. 8.0 [2.3,20.1]

**5 Months**	**RECIST**	24.7 [17.3,47.6] vs. 51.9 [26.6,64.7]	7.6 [4.2,19.6] vs. 11.3 [9.2,12.9]	21.1 [11.0,38.3] vs. 26.4 [9.2,26.5]	7.9 [4.7,19.9] vs. 12.9 [11.3,47.6]	7.6 [4.7,19.8]] vs. 11.3 [9.2,18.1	n/d [24.1,n/d] vs. 26.5 [12.9,n/d]	12.0 [5.0,19.9] vs. 18.1 [10.6,27.4]
	**WHO**	27.7 [17.9,48.0] vs. 21.6 [13.8,26.6]	7.6 [4.2,19.6] vs. 12.9 [7.9,18.1]	21.1 [9.9,38.3] vs. 21.6 [13.0,26.4]	11.3 [4.7,20.9] vs. 12.9 [7.9,18.1]	7.6 [4.7,19.6] vs. 12.9 [7.9,19.9]	n/d [24.1,n/d] vs. n/d [26.4,n/d]	12.0 [5.0,20.9] vs. 18.1 [6.3,27.4]
	**mRECIST**	19.8 [15.6,38.8] vs. 28.8 [19.4,65.0]	5.1 [4.7,7.5] vs. 11.9 [6.9,19.6]	18.1 [9.9,25.6] vs. 24.7 [13.0,26.5]	5.1 [4.7,7.5] vs. 14.0 [7.6,19.9]	5.0 [4.7,6.1] vs. 12.4 [7.9,19.8]	n/d [20.7,n/d] vs. n/d [26.4,n/d]	5.1 [4.7,7.5] vs. 18.1 [10.6,27.4]
	**EASL**	19.8 [15.6,30.4] vs. 28.8 [19.4,51.9]	5.1 [4.7,6.1] vs. 11.9 [6.9,19.8]	18.1 [9.9,25.6] vs. 24.4 [13.0,44.3]	5.1 [4.7,7.5] vs. 14.0 [6.9,19.9]	5.0 [4.7,6.1] vs. 12.4 [7.6,19.9]	n/d [25.6,n/d] vs. n/d [24.1,n/d]	5.1 [4.7,6.1] vs. 18.1 [7.9,27.4]
	**LI-TRA v2018**	18.2 [9.7,26.6] vs. 31.6 [17.3,62.7]	5.0 [2.6,18.1] vs. 10.1 [5.2,19.6]	17.9 [5.2,20.7] vs. 24.4 [11.0,62.7]	5.0 [2.6,18.1] vs. 13.2 [9.2,26.5]	5.2 [2.6,20.7] vs. 10.8 [5.1,19.6]	25.6 [20.7,26.4] vs. n/d [26.5,n/d]	6.3 [2.6,20.9] vs. 13.2 [6.9,27.4]
	**LI-TRA v2024**	18.5 [15.8,39.0] vs. 28.9 [19.7,65.7]	4.7 [2.6,21.3] vs. 9.3 [5.3,19.7]	18.2 [9.8,25.1] vs. 25.8 [13.1,48.4]	4.7 [2.6,23.3] vs. 13.3 [6.4,20.0]	5.1 [2.6,21.3] vs. 10.1 [5.3,19.9]	n/d [21.0,n/d] vs. n/d [24.5,n/d]	5.1 [2.6,23.0] vs. 12.6 [6.4,20.1]

OS overall survival, PFS progression-free survival, TTP time-to-progression, LTP local tumor progression subtype, IDR intrahepatic distant recurrence subtype, RECIST Response Evaluation Criteria in Solid Tumors v1.1, mRECIST modified RECIST, WHO World Health Organization, EASL European Study of the Liver, LI-TRA Liver Imaging and Reporting Data System (LIRADS) Treatment Response Algorithm, n/d non-defined. Data is depicted as median and interquartile range (IQR).

Supplement Table S3. Uni- and multivariate cox proportional hazard model results.

	**OS**			**PFS**			**TTP**		
	**95% CI (exp B)**	**HR**	**p-value**	**95% CI (exp B)**	**HR**	**p-value**	**95% CI (exp B)**	**HR**	**p-value**
**Univariate analysis**									

iBT vs. cTACE/iBT	0.61-1.48	0.95	0.81	0.84-1.85	1.25	0.27	0.89-2.11	1.38	0.15
age	0.98-1.00	0.98	**0.07**	0.98-1.02	1.00	0.99	0.97-1.02	0.99	0.56
male vs. female sex	0.47-1,36	0.80	0.41	0.65-1.71	1.05	0.84	0.54-1.50	0.90	0.69
lesions	0.87-1.34	1.08	0.50	0.93-1.50	1.18	0.18	0.79-1.41	1.05	0.72
sum of lesion diameter	1.00-1.02	1.01	**0.02**	0.99-1.01	1.00	0.79	0.99-1.01	1.00	0.95
non-viral vs. viral cause of cirrhosis	0.56-1.53	0.92	0.76	0.55-1.36	0.86	0.53	0.59-1.56	0.96	0.87
Child Pugh class A vs. B	1.25-5.28	2.57	**0.01**	0.69-2.80	1.39	0.35	0.57-2.75	1.25	0.58
BCLC stage A vs. B	0.89-2.51	1.50	0.13	0.68-1.78	1.10	0.70	0.57-1.69	0.98	0.93
BCLC stage A vs. C	0.90-3.66	1.81	**0.09**	0.89-3.3	1.71	0.11	0.91-1.95	1.82	**0.09**
Alpha-fetoprotein (AFP)	0.99-1.00	1.00	0.28	1.00-1.00	1.00	0.33	1.00-1.00	1.00	0.31
ALBI score	1.65-4.74	2.79	**<0.01**	0.93-2.44	1.51	**0.09**	0.67-1.95	1.14	0.62
AST/ALT-ratio	0.84-1.69	1.19	0.32	0.72-1.62	1.08	0.72	0.61-1.53	0.97	0.88
**Multivariate analysis**									
age	0.95-1.01	0.98	0.29						
sum of lesion diameter	0.99-1.02	1.01	**0.08**						
Child Pugh class A vs. B	0.22-2.65	0.76	0.67						
BCLC stage A vs. C	0.70-4.50	1.77	0.23						
ALBI score	1.38-7.73	3.25	**<0.01**						

OS, overall survival; PFS, progression-free survival; TTP, time to progression; CI, confidence intervals; HR, hazard ratio; iBT, interstitial high dose-rate brachytherapy; cTACE, conventional transarterial chemoembolization.

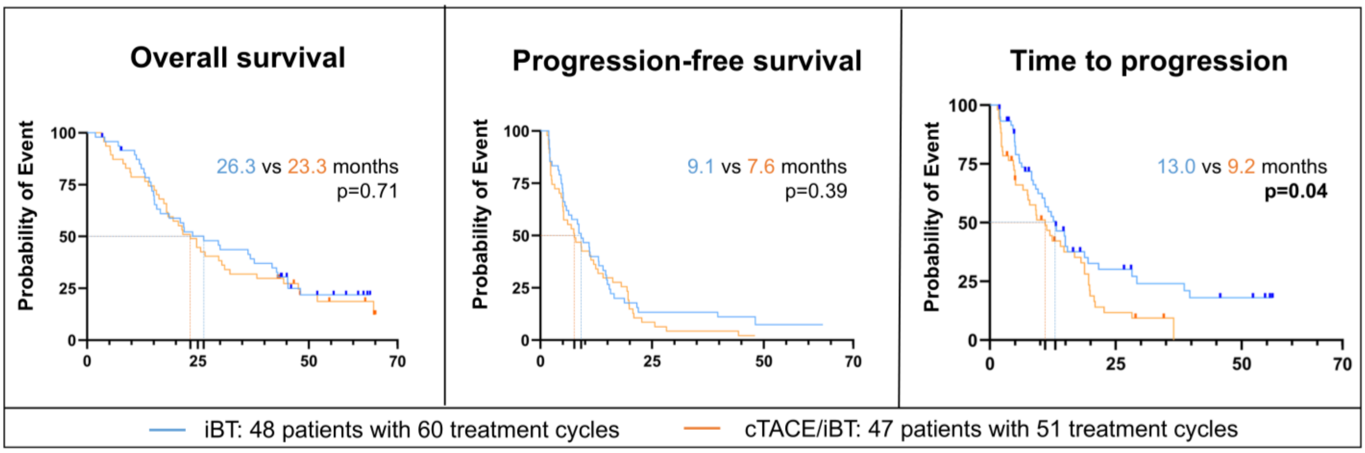

Supplement Figure S1. Overall survival (OS), progression-free survival (PFS) and time to progression (TTP) in patients following interstitial brachytherapy (iBT, blue line) alone or with a combined prior conventional transarterial chemoembolization (cTACE/iBT, orange). Dashed lines show 95% confidence intervals. Kaplan-Meier analyses reveal no significant differences for OS and PFS, but for TTP.

Disclaimer/Publisher's Note: The statements, opinions and data contained in all publications are solely those of the individual author(s) and contributor(s) and not of MDPI and/or the editor(s). MDPI and/or the editor(s) disclaim responsibility for any injury to people or property resulting from any ideas, methods, instructions or products referred to in the content.
